# Socio-economic inequalities in modern Russia and their perception by the population

**DOI:** 10.1186/s40711-020-00124-9

**Published:** 2020-07-01

**Authors:** Svetlana Mareeva

**Affiliations:** 1 Center for Stratification Studies, National Research University Higher School of Economics, Moscow, Russia; 2 Institute of Sociology FCTAS RAS, Moscow, Russia

**Keywords:** Inequality, Poverty, Income concentration, Public perception, Fairness, Russia

## Abstract

This paper focuses on the objective situation regarding inequalities and their subjective perception by the population in Russia in recent years. It is shown that socio-economic inequalities are currently perceived by the population as excessive and illegitimate, and the gap between expectations and social reality has led to growing requests for “leveling” being made to the state. This analysis of the perception of social inequalities is carried out against a background of the objective situation with inequalities that is characterized by the equalization of incomes in the middle layer of society and simultaneously by the growing gap between the top and the rest of the population. Key challenges and crossroads that the state faces in terms of developing socio-economic policies aimed at reducing inequalities are identified.

## Introduction

Inequalities represent a highly sensitive issue in the current stage of global development. The problem of socio-economic inequalities has consistently been at the foreground of academic and public discussions following the 2008 financial crisis; however, concerns about the consequences of high and, worse still, rising inequality were voiced in academia before the crash. In particular, economists point to growing (or, at least, not decreasing) inequality in income and wealth, as shown by a whole range of publications on this issue in recent years (Stiglitz 2012; Piketty 2014; Atkinson 2015; Milanovic 2016). Sociologists, in turn, focus on the persistence of ascriptive causes of inequality and the emergence of new dimensions while the old ones remain in place, despite the declared goal of reducing inequality by means of socio-economic policy (Grusky 2011). Concerns about the problem of inequality are not confined to academic circles—for example, reducing inequality within and among countries was included in the UN Sustainable Development Goals in 2015. A whole range of international organizations’ reports and conferences has also tackled the issues of global inequality in recent years (EBRD 2017; Hardoon et al. 2016; World Bank 2016).

A separate but important issue is the population’s perception of social inequalities and their acceptable depth. The demand for reducing inequalities can develop in conditions in which they are excessively deep, or when inequalities or their sources are regarded as illegitimate and unfair. The negative consequences of the public’s view of inequalities as being too high, having unjust grounds, and on the whole conflicting with the “ideal” social model, can have far-reaching social implications—they can generate social tension, provide a basis for delegitimization of the government in the eyes of the public, and contribute to demand for a revision of the social contract with the state (Bussolo et al. 2019). On the other hand, the perception of inequalities as meritocratic can become a resource for economic development, a driver of the population’s investments in human capital and increased productivity. The subjective perception of inequalities can be considered as part of a broader discussion on fairness (Sztompka 2015) and the necessity of taking into account people’s subjective views for the evaluation of social prosperity and progress (Stiglitz et al. 2014). Although studies show that people can be wrong in their assessment of the depth of objective income inequality and their personal positions on the ladder of inequality (Gimpelson and Treisman 2018), subjective estimates as such are definitely important, as they can serve as a precondition for social actions and the choice of certain behavioral strategies on the micro-level. Besides, subjective perception of inequality can affect even the general direction of academic and social discourses (Piketty 2014). In this case, however, we will not touch upon this subject.

The severity, factors, and consequences of objectively existing inequalities are actively discussed by Russian scholars as well, with economists and sociologists tackling these topics from different angles (Anikin and Tikhonova 2016; Kapeliushnikov 2016; Ovcharova et al. 2016). However, comparatively less attention is paid to the issue of the population’s perception of inequalities (Gimpelson and Monusova 2014) and of the social structure, although some studies in this domain have been conducted (Kosova 2016; Mareeva and Tikhonova 2016).

In this paper, we will analyze the population’s subjective perception of inequalities against the background of the objective monetary inequality in Russia, and identify major challenges and choices for socio-economic policy aimed at reducing inequality. It may be interesting to compare the example of Russia with the experience of countries in Western and Eastern Europe, as well as the BRICS countries (Brazil, Russia, India, China, and South Africa). All of them are facing the problem of social inequalities and the challenge of reducing them, but responses can vary, depending on the different norms and values of the population and the different types of public demands on the government, as well as on resources and the priorities of social policy. Moreover, even the understanding of the problem of inequality may vary depending on the socio-economic stage of the county’s development and dominating norms governing the relationship of the population with the state. With a formally universal global agenda, the fight against inequalities in practice may require measures such as supporting the most disadvantaged population, providing a certain minimum standard of living for the entire population (hence the idea of a universal basic income), reducing the concentration of income and wealth in the hands of the minority, decreasing gaps in wages for jobs that require similar qualifications, and narrowing gaps in access to basic social services (which exist due to infrastructural problems or corruption).

We primarily focus on the discussion of the state of socio-economic inequalities in Russian society and how they are perceived by the population, trying to explain the factors of divergence between objective and subjective measures of inequality. In this discussion, we turn to the evidence from different empirical sources. The first one is official statistics collected and presented by the Federal State Statistics Service (https://eng.gks.ru). The second is microdata from a widely known all-Russian survey, the Russia Longitudinal Monitoring Survey conducted by the Higher School of Economics (RLMS-HSE)^1^. Finally, we also use microdata from the monitoring carried out by the Institute of Sociology, Federal Center of Theoretical and Applied Sociology of the Russian Academy of Sciences (IS FCTAS RAS)^2^.

Due to the obvious constraints, we rely on the data collected before the changes in the socio-economic realities of life that occurred due to the coronavirus pandemic and the dynamics of oil prices in 2020. However, under these conditions, it is even more important to analyze the situation with objective inequalities and their perception by the population at the “starting point” of the crisis, since the deterioration of socio-economical and health-related conditions will most probably lead to intensification of the negative trends concerning both the objective state of inequalities and their subjective perception by Russians.

## Poverty and inequality in Russia: objective situation

Reducing inequality is one of the common points of the Russian socio-economic agenda and is often tied in with the goal of alleviating poverty. However, bringing down the level of poverty does not automatically imply less inequality, although it can contribute to this by bridging the gap between the lower and lower-middle strata.

There are three major methodological approaches to identifying the poor—absolute, relative, and subjective. Within the absolute approach, poverty is understood via comparison of an individual or household income to a “poverty line” determined by experts: if income is lower than the poverty line set in quantitative terms, the individual or household is considered to be poor. The relative approach identifies groups within the population that are not able to maintain a standard of living that can be considered typical for a given society, by either monetary (hence the relative poverty line, usually set as a certain share of median income in the country in the range of 0.5–0.7) or non-monetary (deprivations in different spheres of everyday life) measures. The subjective approach deals with the socio-psychological perception of one’s position in society.

For the aims of socio-economic state policy and official statistics, *an absolute approach to poverty is officially used in Russia.* Individuals are categorized as poor if their income is below the official minimum subsistence level established in each region for three categories—working-age population, children, and pensioners. In the second quarter of 2019, the subsistence level for the country was set at RUB 11,185/person (that is, approximately USD 170 by nominal exchange rate or USD 460 by the purchasing power parity (PPP) exchange rate set by the World Bank^3^). As a result, 14.3% of the population were considered to be poor by official statistics.

The situation with poverty has noticeably changed in Russia during the last two decades. *The socio-economic transformation of the 1990s resulted in significant deterioration of the quality of life and contributed to mass poverty of the population during this period, including among educated and qualified workers*. According to official statistics, one third of the population—33.5%—were poor in 1992, so poverty was basically a norm for society at that time. Due to the external, structural reasons for their poverty (unemployment, non-payment of salaries and social benefits, minimum level of social benefits), *the public attitude towards the poor was generally sympathetic*. However, as the country’s economy and population adapted to the new reality, the situation regarding poverty also started to change. *Starting from the early 2000s, the percentage of people classified as poor has been shrinking* due to the growth of social support (including pensions) and the elimination of mass non-payment of salaries, etc. (Table 1). The latest crisis halted this trend, but even amid the crisis, mass poverty is no longer the norm for the Russian population.

**Table 1:** Russian population with income below the minimum subsistence level, 1992–2019

Year	Number of people classified as poor (in millions)	Percentage of the total population classified as poor
1992	49.3	33.5
1994	32.9	22.4
1996	32.5	22.1
1998	34.3	23.4
2000	42.3	29.0
2002	35.6	24.6
2004	25.2	17.6
2006	21.6	15.2
2008	19.0	13.4
2010	17.7	12.5
2012	15.4	10.7
2014	16.1	11.2
2016	19.5	13.3
2018	18.9	12.9
2019 (1st quarter)	20.9	14.3

Source: Official data of the Russian Federal State Statistics Service (see: http://www.gks.ru/free_doc/new_site/population/urov/urov_51g.doc, accessed January 5, 2020)

Therefore, during the last two decades, *Russia has transformed from a mass poverty society to a mass middle-income society.* Moreover, from a group that is only different from the rest of the population by income level and consumption limitations, the poor are turning into an isolated, self-reproducing social group with a different socio-professional composition, lower level of education, lower life chances, and higher risks in everyday life (Tikhonova and Mareeva 2016; Tikhonova and Slobodenyuk 2015).

Another illustration of this trend is the dynamic of the poor and middle classes^4^ in Russia according to international monetary thresholds. One often-used approach is that of the World Bank (World Bank 2014), which sets a daily income of USD 5 as the poverty line and USD 10 as the bottom line for the middle-income range. Those who live on between USD 5 and 10 are considered to be vulnerable to falling into poverty. Applying these thresholds to RLMS-HSE data (using the World bank’s PPP calculation) reveals an extremely low proportion of Russians (just 1.5–2.5%) were classed as being in poverty even during the 2008 economic crisis, while the share of those vulnerable to poverty is around 10%.

Using this approach, the overwhelming majority of the population (over 87% in 2017) falls within the middle class, having a daily income exceeding USD 10 (Mareeva and Lezhnina 2019).

Again, this situation highlights fundamental changes in Russian society in the last two decades. The World Bank’s calculations show that only slightly more than a quarter of the Russian population (27%) belonged to the middle class defined by this criterion in 2000, but this share had increased to 60% by 2010; survey microdata show that the expansion of the middle class continued further, bringing it to almost 90% of the population in recent years. That means that Russia has already moved away from the minimum physical survival standard for the population that these ranges are based on, has solved the problem of extreme poverty, and, according to the World Bank, is currently an “upper-middle-income country”^5^.

As a result, *the problem of poverty has been transformed in the public perception as well.* The proportion of people who have someone poor in their immediate circle and feel sympathetic towards them has declined (Tikhonova and Mareeva 2016), and *the structural factors of poverty (unemployment, inefficient system of social support, etc.) have become much less significant in the public consciousness, although they so far still outweigh individual factors (such as alcoholism, drug addiction, and laziness), the importance of which has increased in subjective assessments.* For example, according to the IS FCTAS RAS data, the share of the population who see the non-payment of salaries and delayed pensions as one of the main factors of poverty in Russia declined from 46.8% in 2003 to 23.4% in 2015; for long-term unemployment, the figure fell from 41.2 to 31.3%; and for insufficiency of social benefits, from 37.1 to 24.9%. At the same time, laziness as a factor of poverty has gone up in general ratings of poverty factors in the public consciousness, from 22.6 to 30.5%.

All of these trends (the decrease in the number of poor people, changes in their socio-professional composition, and re-defining of the poverty factors in public opinion) explain why the Russian population is currently concerned not so much about poverty as about inequalities and, therefore, gives priority to fighting inequalities but not poverty (according to 2018 data from IS FCTAS RAS Monitoring, 41.2% absolutely agreed, while only 15.6% disagreed; the rest found it difficult to choose a priority).

Of course, the poverty problem cannot be left off the socio-economic agenda. In fact, the Executive Order of the President of Russia “On National Goals and Strategic Objectives of the Russian Federation through 2024” (known as the “May Decrees”) signed in 2018 aims to cut poverty in half, though the feasibility of this aim is a topic for discussion. Therefore, one of the socio-economic policy challenges is developing and demarking measures designed to fight poverty, as opposed to measures against inequality that would address the demands of the population and reduce social tension. The course of state socio-economic policy on increasing targeted social assistance that was chosen in recent years can help with reducing poverty, but does not fully correspond to the public demands on reducing inequalities or stabilizing the positions of the lower-middle and middle strata. Finding the balance between measures aimed at fighting poverty and those aimed at reducing inequalities (not only between the most disadvantaged section of the population and others) is one of the challenges for the state.

Turning to the objective state of affairs regarding monetary inequality in modern Russia, *income inequality estimates associated with income distribution across the population and typically measured with the Gini coefficient indicate that the level of inequality in the country is high, especially compared with Western European countries, but not extreme*.

As can be seen from the dynamics of the Gini coefficient estimated by the Russian Federal State Statistics Service, from 1994 to 2007, overall inequality in the country increased (0.409 to 0.422). After that, it began to decline but only slightly, reaching 0.409 in 2017 (and 0.411 in 2018, according to preliminary estimations) (Table 2).

**Table 2:** Gini coefficient in Russia, 1994–2017

*Year*	*1994*	*1995*	*1996*	*1997*	*1998*	*1999*	*2000*	*2001*	*2002*	*2003*	*2004*	*2005*
Gini coefficient	0.409	0.387	0.387	0.390	0.394	0.400	0.395	0.397	0.397	0.403	0,409	0.409
*Year*	*2006*	*2007*	*2008*	*2009*	*2010*	*2011*	*2012*	*2013*	*2014*	*2015*	*2016*	*2017*
Gini coefficient	0.415	0.422	0.421	0.421	0.421	0.417	0.420	0.419	0.416	0.413	0.412	0.409

Source: Official data of the Russian Federal State Statistics Service (see: http://www.gks.ru/free_doc/new_site/population/bednost/tabl/1-2-2.doc, accessed January 5, 2020)

**Fig. 1:**
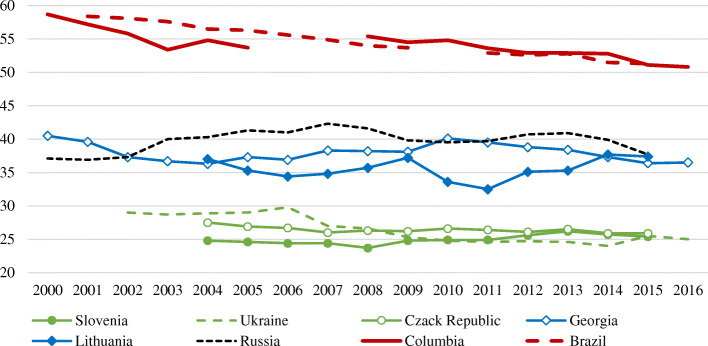
Gini coefficient in Russia and other countries, 2000–2016. Source: Gini index, World Bank estimate (see: https://data.worldbank.org/indicator/SI.POV.GINI, accessed January 5, 2020). The author is thankful to E. Slobodenyuk for graph visualization

Data on the Gini coefficient provided for Russia by the World Bank is different from official Russian statistics, as the World Bank does not perform income data imputations. In an international context, according to the World Bank, the upper border of inequality is set by Latin American countries (Fig. 1), while Russia sets the upper border for European countries.

In other words, in a global context, Russia is characterized by the middle level of income inequality, but among the developed countries, its income inequality level seems to be rather high. Moreover, according to World Bank estimates, a Gini index of 38–40% means an excessive level of inequality that negatively affects economic growth, and income inequality in Russia is on the border of this level. However, studies have shown that the effects of inequality on economic growth differ depending on the income level in the countries under consideration: in low-income countries, rising inequality negatively affects economic growth, but in high-income countries, there is no negative effect (Barro 1999).

If the dynamics of monetary inequality are measured by the ratio of the income growth of the least advantaged groups to that of the general population, then the World Bank data show that inequality in Russia has been decreasing in recent years, as the income of the bottom 40% of the population has been growing faster than the mean income in the country as a whole (World Bank 2016).

Another approach to measuring monetary inequality, but with a more sociological focus, is the analysis of income stratification—defining different income groups on a scale of “low income to high income.” Models of income stratification can be constructed in frameworks of absolute or relative approaches. One example of an absolute approach to income stratification, as presented above, is the model used by the World Bank, according to which income stratification in Russia is much closer to that of Western Europe, where most of the population belongs to the “middle class” and the relative number of poor is small and extreme poverty virtually non-existent. However, while the configuration of the income structures is similar in terms of the proportions of different income groups, the absolute levels of income of middle class representatives in Russia and Western Europe are quite different.

**Fig. 2:**
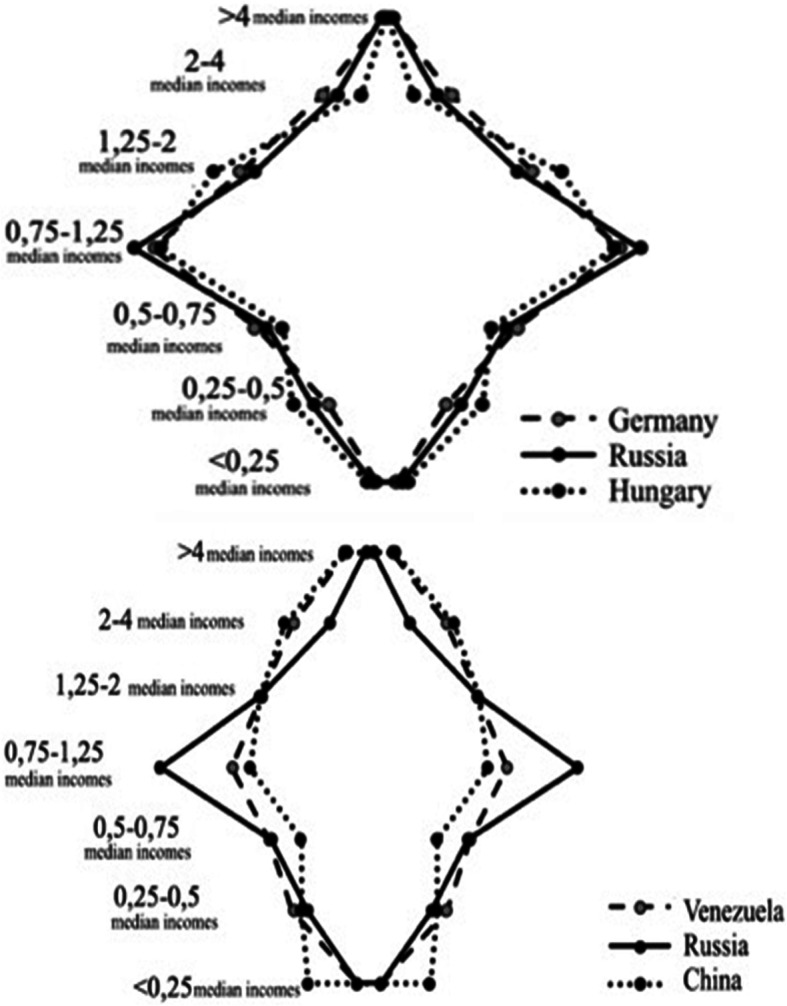
A graphical representation of income stratification in Russia vs. China, Venezuela and Germany, Hungary. Source: Tikhonova 2018a; graphs are based on ISSP data (see http://w.issp.org/menu-top/home/)

If we turn to the income stratification model in traditions of relative approach, where income groups are defined based on the ratio between their representatives’ income and the country’s median income (see more on the methodology in Mareeva and Lezhnina 2019), then *the model obtained for Russian society is also much closer to those of developed countries, marked by a broad middle stratum and the absence of extreme poverty, as opposed to the models typical for other BRICS countries or Latin America* (Fig. 2)*.*

The median-income group (the population with an income between 0.75 and 1.25 times the national median) currently dominates in the income structure of Russian society, bringing it closer to the models in countries like Hungary or Germany. At the same time, however, the income level and the respective standard of living among the Russian middle strata are quite modest, though far from the level of survival: according to the RLMS-HSE data, only 1/5 of this group are satisfied with their material position, less than 1/4 have any savings, and most of the representatives of the median-income stratum do not have any opportunities to improve their housing situation, save money for large purchases, or spend family vacations abroad. At the same time, over 2/3 of the group have a computer or laptop in the household, as well as a washing machine and TV, and over 4/5 have personal cellphones.

**Fig. 3:**
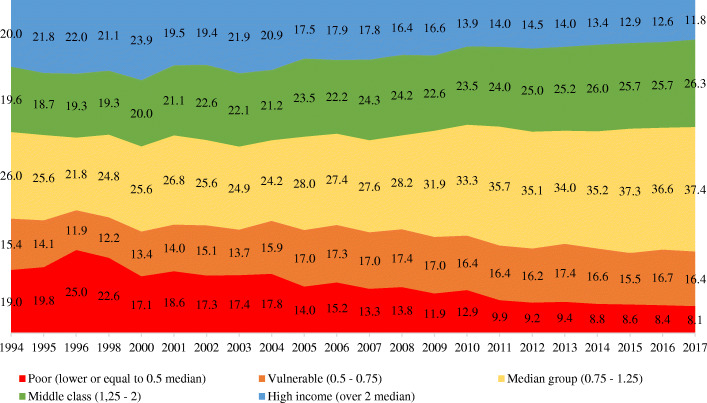
Relative income stratification dynamics in Russia, 1994–2017, percentage of income groups. Source: RLMS-HSE data, calculations by the author

*Income group dynamics in the last two decades show a notable expansion of the “middle” stratum and a contraction of the high-income and low-income groups* (Fig. 3). The median group, according to calculations on the RLMS-HSE database (defined with the use of the OECD equivalence scale to account for household size), has increased from 26.0% of the population in 1994 to nearly 37.4% in 2017. However, its growth was caused not only by a decline in the low-income population but also by a notable narrowing of the prosperity zone^6^: The poor population, with an income less than 0.5 times the median, dropped from 19.0% in 1994 to 8.1% in 2017, while the high-income population, with an income more than twice the median, fell from 20.0 to 11.8%. Therefore, according to the data on mass strata of the population, *the last two decades were characterized by a tendency towards the equalization of incomes and an increasing proportion of the population with incomes close to the median value*—in other words, by a growing number of people who are living in roughly the same conditions and have similar living standards that reflect the general living standard of the country as a whole, while being quite different from the typical conditions of the groups that are at the top and bottom of the income scale. That trend can be characterized as *averaging out of the incomes in the middle*.

All of the indicators described above provide a static picture of inequality. However, this can be altered by individuals changing places within the income distribution range over time—in other words, by different patterns of intergenerational and intragenerational mobility. *Data show that while according to the income stratification model Russia is closer to developed than to developing countries, which are characterized by a high share of groups with median and middle incomes, in regard to the income mobility models, Russia is closer to developing countries, which are characterized by relatively high-income mobility and a lesser degree of persistent inequality* in the medium-term. Analysis of relative individual income mobility (movement between income quintiles) over a 4-year interval shows that in comparison with the OECD countries, income mobility in Russia is rather high. In 2014–2017, individual income mobility for the working-age population was as follows: 41.8% stayed in the same income quintile, 29.4% were characterized by upward mobility, and 28.8% experienced downward mobility. In OECD countries, on average, half of the population stayed in the same quintile, nearly quarter of the population moved up one quintile or more, and another quarter of the population went down one quintile or more (OECD 2018). Furthermore, the situation in Russia, as in other countries, is marked by a “sticky floor” and a “sticky ceiling”—those who steadily remain in the lowest or highest income quintiles. Among those who were in the first quintile in 2014, 58.9% remained there in 2017; for the fifth quintile, the respective share was 54.3%. On average across OECD countries, these shares were similar for the bottom quintile (around 60%), but reached 70% for the top one. Over a 9-year interval, the “sticky floor” effect in Russia is also comparable to the OECD countries, but the “sticky ceiling” effect remains smaller. Therefore, *compared to the averaged OECD data, Russia is characterized by having a smaller scale of stable well-being among mass strata of population.*

It would appear that such transformation dynamics of the income stratification model associated with increasing equality among the general population and rather high-income mobility should ease social tension over inequalities. In practice*, however, the problem of inequality remains pressing in the public consciousness. In fact, it has not only failed to recede into the background during the latest crisis but has become even more acute,* as will be demonstrated below. One of the possible reasons for this is the *growing gap between the select few at the top and the rest of the population* that cannot be seen when measuring inequality in mass strata. Measuring inequality through income or wealth concentration shows that on a global scale, *Russia is one of the leading countries in terms of income concentration* among the top 1–5% of the population, *and wealth concentration levels are even higher* (Credit Suisse 2019). For example, according to the World Income Database (WID; see Alvaredo et al. 2018), in 2016, the share of national income per top 10% of the population with the highest income was 37% in Europe, 41% in China, 46% in Russia, and 47% in the United States and Canada. Differences are even more distinct for the top 1% of the population who account for 20–22% of total income in Russia, which roughly corresponds with the same indicator for the US, and significantly higher than in China and the other transitional countries of Eastern Europe, where it is in the range of 10–14% (Novokmet et al. 2017).

The growth of inequality in Russia after 1980 is considered by the WID researchers to be high, as it is in North America, China, and India, while the growth of inequality in Europe is seen as moderate. The extreme concentration of income and wealth in Russia is attributed by the researchers to the specific nature of the chosen transition path to a market economy, in particular, “shock therapy” and the voucher-based privatization that was carried out very fast and which took place in a “legislative and institutional vacuum” (Novokmet et al. 2017, p. 37), leading to consolidation of ownership in the hands of a select few. In the newly established institutional environment, political ties proved to be more important than managerial and entrepreneurial talents (Suisse 2012) or even the rule of law, and major owners had a great influence on the “rules of the game,” including the judicial system. In contrast, in other countries of Eastern Europe, a different institutional context evolved, with a more significant role for the law, protection of property rights, and more successful creation of market economy institutions, resulting in a different pattern of inequality.

Therefore, *the objective dynamics of inequalities in Russia in recent years are non-linear: the reduction in poverty and some equalization of incomes in the middle section of society are simultaneously connected with by the growing gap between the top and the rest of the population.*

Some specifics of socio-economic policy in contemporary Russia and the redistributive effect of tax-benefit policies on inequality should be mentioned in this regard. The Russian welfare system currently represents a mix of elements from state socialist and neoliberal models (Popova et al. 2018). The state-paternalist model underwent demolition during the radical liberalization in the 1990s, but a revival of welfare statism happened in the mid-2000s (Cook 2010), when social policy was declared as one of the national priorities—however, it was not fully reflected by the actions and decisions taken (Kainu et al. 2017). According to estimates, the Russian welfare state achieved a moderate reduction in inequality through tax-benefit policies by international standards, with most redistribution occurring through pensions. That is due to several factors, including the low share of spending on social assistance targeted to low-income groups^7^, a flat rate of 13% for individual income tax and regressive indirect taxes (Popova et al. 2018).

The series of new national projects initiated in 2018^8^ are aimed, in part, at reducing non-monetary inequalities in healthcare, education, and housing; however, the aim of reducing monetary inequalities is not explicitly declared. The “May Decrees” mentioned above put into focus poverty and the growth of real incomes, but not inequality. Moreover, the implementation of these national projects might be hindered by the unfolding of the new epidemiological and economic crisis.

## Subjective perceptions on inequality in Russia

Let us now turn to the reflection of the objective picture of inequalities in the subjective perceptions that have formed in the public consciousness. We have already mentioned that *the problem of inequalities, unlike poverty, did not fade into the background, and, on the contrary, has become even more disturbing for the population.* In particular, only 1.5% of the population did not feel any acute inequalities in modern Russia in 2018, and a mere 9.3% said they themselves were not affected by any inequalities (Table 3). Income inequality topped the list of the most painful inequalities—83.8% of the population found it to be the most distressing for society as a whole, while 69.4% struggled with it personally. Non-monetary inequalities, especially those related to basic living standards such as healthcare and housing, were also acutely felt by the population. They were followed by a group of inequalities that had to do with social mobility opportunities—access to good jobs and education, and the lack of an equal head start among children from various social strata.

**Table 3:** Most painful inequalities in the eyes of Russians, 2018, %

Types of inequality	Painful for society	Painful for respondents themselves
Income inequality	83.8	69.4
Inequality of access to medical care	***69.6***	***51.2***
Inequality of living conditions	64.0	***36.0***
Inequality of access to good jobs (*for working population*)	51.9	37.5
Inequality of access to education	***47.7***	***22.5***
Inequality of opportunities for children from different social groups	32.6	19.0
Inequality of available leisure activities	***22.4***	***27.0***
Inequality of property ownership	19.5	15.6
Inequality in social capital	8.8	10.1
None	1.5	***9.3***

Source: IS FCTAS RAS data, calculations by the author. The positions where the increase since 2015 has exceeded 5% are indicated in bold cursive

In recent years, *the overall rating of the most significant inequalities in Russia affecting the lives of individuals and society at large has remained the same in the public perception, but their perceived severity has grown.* As for society in general, Russians increasingly mention the problem of inequality in access to healthcare and education. These two types of inequalities are directly related to human capital, and such dynamics in this respect is particularly disturbing.

It is not only the acuteness but also the general fairness or unfairness of inequalities in the public perception that is important, and in this regard, the situation in Russia is even more troubling. According to IS FCTAS RAS data from 2018, around 50% of Russians believe that the current system of private property distribution in Russia is unfair, and another 41.6% partly agree with this. Assessing how abilities and qualifications are rewarded in Russia today, only 15.7% agree that people get decent pay for them, 40.0% partly agree with this, and 44.3% do not agree with this statement at all. At the same time, assessing their own situation, Russians are even more critical—87.2% fully or partially agree that they receive significantly less than they deserve. Therefore, *both the distribution of private property and the remuneration system that currently exist in Russia are considered to be unfair by the majority of the population*, irrespective of people’s own situation, although the perception of their personal situation varies among representatives of groups with different income levels or life chances. This means a universal, shared demand among the population for reducing inequality and achieving fairness in Russian society.

Prevailing public views on the causes of wealth and prosperity in modern Russian society also contribute to this demand—the majority of the population believes that it mostly comes down to luck and social capital rather than to personal skills and efforts. This view is shared by representatives of both low-income and high-income groups, though an increase in income level brings an increase in the shared notion on the meritocratic foundations of well-being (Table 4).

**Table 4:** Perceptions on the causes of well-being in Russia by population, 2018, %

Causes of well-being in public perception	All population	Low income (< 0.75 median income in the country)	High income (> 2 median income in the country)
High level of education and qualifications, effective work, efforts	38.5	31.7	51.1
Luck and social capital	61.5	68.3	48.9

Source: IS FCTAS RAS data, calculations by the author

A serious mismatch between reality and the expectations of the population regarding social inequalities is evidenced by the *apparent gap between the “ideal” and “real” models of modern Russia’s social structure in the public consciousness*. While describing the real models of the Russian social structure, the population usually chooses high-inequality models (1 and 2 in Table 5)—a “pyramid” (44.3%) or a model with the chosen few at the top and the masses at the bottom (30.6%). Models characterized by a mass middle stratum—with smaller upper and lower strata or without them at all (3 and 4 in Table 5)—are chosen only by a quarter of Russians when describing the existing reality. At the same time, speaking of the ideal model, over half (53.9%) of the population chooses a society of social homogeneity as the ideal social model, while models without a mass middle stratum are considered ideal only by 26.5% of the population.

**Table 5:**
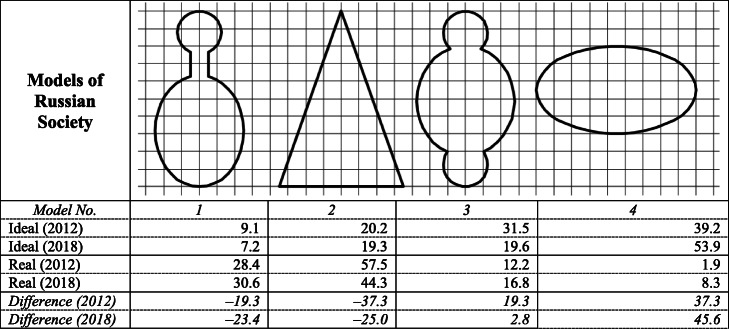
Real and ideal models of social structure for Russian society in Russians’ opinion (age 18–55), 2012-2018, %

Source: IS FCTAS RAS data, calculations by the author

The choice of a socially homogeneous society as the ideal model has only become dominant only in the last few years (just 39.2% opted for it in 2012), which reflects an important shift in the public consciousness. Nowadays, *models with a high degree of inequality continue to be seen by Russians as a reflection of the real, but not ideal, structure of society, while social equality increasingly reflects the “unrealizable ideal,” demonstrating the growing desire to decrease inequalities in Russian society.*

This imbalance affects the perception of the country’s situation and prospects—Russians who are acutely aware of the gap between the ideal and real models (those who chose model 1 or model 2 as the reality for Russian society, but models 3 or 4 as the ideal) not only have lower trust in political institutes and share more pessimistic views about Russia’s future development, but are also much less optimistic about the country’s chances of achieving different development goals, including those aimed at reducing inequality; it is especially important since this group comprises more than half of the population (57.2%).

It is worth noting that Russians expect social fairness to be guaranteed by the state—not only because it is the state’s “fault,” but also because *the role of guarantor of social justice in the Russian socio-cultural model is consistently assigned to the state. The state has to be the actor balancing the interests of various social groups, as opposed to the separate groups defending their interests while competing with each other* (for more on this, see Tikhonova 2018b). At present, a dominant majority of the population (74.5%) agree that the federal government should be responsible for the fair distribution of wealth among the population, and 11.1% put this responsibility in the hands of regional governments.

Furthermore, the majority believes that the state is currently failing in this task. The data from the latest European Social Survey wave^9^ indicate that *Russians are generally in the same “borders” as most Europeans in terms of tolerance for income inequalities and inequalities in living standards; however, it is Russians who are most critical of and negative about the government’s ongoing efforts to reduce income inequality*. More than 40% of the population do not agree that the system of social support in the country helps reduce inequalities, while only 1/4 agree with that statement. Addressing this need while taking into consideration the heterogeneity of various social groups’ specific problems is another important socio-economic policy challenge.

Yet the challenges of socio-economic policy aimed at social inequalities have to do not only with the problems but also with taking advantage of the potential and associated resources of inequalities (and their perception by the population). This potential can be still found in Russian society, although it has been decreasing in recent years.

First, *when speaking of equality, the majority of Russians still mean equality of opportunities and not income equality* (the ratio of these models’ supporters is now 58.7 to 41.3). This ratio has been quite stable over the past decade, although compared to 1995 (when it was 25/75), the share of those who choose equality of income as opposed to equality of opportunity has increased. This may reflect disappointment in the “rules of the game” that do not contribute to the consolidation of justifiable grounds for inequality in the eyes of the population.

Second, opportunities for using the productive role of inequalities are also indicated by the fact that the tolerance towards “fair” inequalities, based on legitimate grounds, is still largely supported in the public consciousness. For example, the salary of a highly skilled professional in the view of Russians should be on average nearly five times higher than the average salary in the country, which means a fairly high income differentiation in society at large. The demands of Russians for equality cannot be reduced to the desire to “take away everything and share it equally”—*Russians are willing to tolerate considerable income inequalities if they believe there are legitimate grounds for them.*

Today, a greater share of Russians tend to agree, rather than disagree, that those who work faster and more efficiently should receive more than those who occupy the same positions but work less efficiently (62.4% agree with this and just 6.4% do not agree), as well as the fact that income inequality is fair if everyone had equal opportunities (50.7% and 15.1%). Higher incomes for those who received a higher level of education are considered fair by 45.2% and unfair by 10.0%. Thus, *work efficiency and qualifications under conditions of equal opportunities for all continue to remain legitimate factors in the eyes of Russians for the formation of monetary inequalities*. At the same time, various professions are not likely to be perceived by Russians as such a fair foundation—they are considered a fair basis for inequality by only 25.2%, and unfair by 34.0%.

On the other hand, a rather high percentage of those who partially agree and partially disagree with the fairness of the abovementioned causes of inequality demonstrates that these notions are becoming vaguer with time. Moreover, *tolerance for all causes of inequality has decreased in recent years.* In 2013, higher wages for those who work faster and more efficiently seemed fair to 73.9% of the population (2018, 62.4%), and for those who had a higher level of education, the figure was 62.7% (2018, 45.2%). Income inequality arising in the conditions of equal opportunities was considered fair by 64.5% of Russians (2018, 50.7%), and different professions were seen as a fair basis of income inequality by 46.8% (2018, 25.2%). Therefore, a significant decrease in tolerance can be seen in respect to all the foundations of inequalities that seemed fair to the majority of the population in 2013.

This also correlates with changes in the population’s views on the ideal and real social structure models mentioned above*—inefficiency or lack of “rules of the game,” which are seen as fair in the normative model but are not observed in practice, results in a growing demand for universal “leveling out.” Such processes are indicative of a diminishing potential for the use of legitimate inequalities as an effective driver of country’s development.*

Even more ambiguous is Russians’ attitude to various outcomes of monetary inequalities—the possibilities of securing a better standard of living and quality of life for those with higher income. Thus, the population is quite tolerant of better housing for those who have higher incomes (46.2% agree that this is fair, against 13.8% who feel it is unfair) and for better education for their children (37.4% and 21.6%, respectively). With regard to higher pensions for those who have higher salaries, the split is closer (30.6% and 25.3%), and as for better medical services for those with higher incomes, the percentage of those who agree with the fairness of this opportunity is much lower than the percentage of those who disagree with it (22.6% and 39.6%). Apparently, paid education for children (with the option of free elementary, secondary, and higher education for all that is still dominant in the Russian educational system) is accepted by a significant part of the population not as an additional opportunity for social mobility and a more successful head start, but as one of the consumption-related practices, which leads to a more tolerant assessment of this manifestation of inequality; in contrast, with regard to inequalities in the vital sphere of medical care (taking into account the aggravation of this problem in the eyes of Russians in recent years), the population is not tolerant at all.

Dynamics in this regard also demonstrates a decrease in tolerance to manifestations of inequalities in different areas of life: for example, in 2012–13, 51.6% considered it fair that people with higher incomes could enjoy better housing (2018, 46.2%), 49.8% agreed with the fairness of opportunities for better education for children from wealthy families (2018, 37.4%), 48.4% considered higher pensions for those with higher salaries to be fair (2018, 30.6%), and access to higher quality medical services for those with higher incomes was regarded as fair by 27.3% (2018, 22.6%).

Therefore, over the 5 years to 2018, *there has been a decrease in tolerance both of the foundations of inequalities that previously seemed legitimate to the population and of various manifestations of non-monetary inequalities based on income inequality—probably since the meritocratic grounds of monetary inequality are becoming more and more questionable.* In general, all of these indicators still show some resource for the use of “productivity” of inequalities and their stimulating role in Russian society, but the dynamics indicates that this potential is gradually decreasing.

## Conclusion

The population’s perception of poverty and inequality reflects the objective trends of the evolution of respective phenomena in Russia from the early 2000s to the beginning of the current economic crisis—a decrease in the share of absolute poverty and the changes in its profile, and growing dissatisfaction with social inequalities. While the problem of poverty fades into the background of public consciousness, Russians continue to be very sensitive to inequalities, despite some of the objective trends that might have mitigated the problem—for example, the expanding middle stratum and high-income mobility that has characterized development of Russian society in recent years.

That is due to several factors: first, to the aforementioned growing gap between the very top of society and the rest of the population. Second, Russians are concerned not so much about income inequality per se (they even consider it to be necessary to a certain degree) as about the unfairness of its causes and non-monetary aspects in modern Russia. They express a need not for an overall “leveling out” of income but for ensuring equality of opportunity under which different levels of income will be based on legitimate factors—education and skills, job performance, etc. Growing equalization among broad segments of the population and the shrinking high-income segment (within the mass population) does not meet the needs of the most educated and qualified Russians, as their risks of losing their positions are growing. Besides, high-income mobility, which is determined not only by socio-professional and socio-educational factors, but is largely due to socio-demographic factors (such as having children or pensioners in the household), becomes not a performance driver and a manifestation of the possibility of potential success for everyone but an indicator of instability and a trigger of social tension. This poses an additional challenge for socio-economic policy, with the need to ensure an institutional environment that will help stabilize the average Russian’s income (measures that contribute to a more secure position in the labor market, offset temporary situational income drops, etc.).

Socio-economic inequalities are a serious challenge for the state, which the population sees as the main actor in solving this problem. The imbalance between the “ideal” and “reality” in this case might be dangerous in terms of a decrease in confidence in the government and a reduction in its “corridor of possibilities” to act against that challenge; it is also important to note that the problem of inequalities is recognized by representatives of both the most and least prosperous groups.

There is also a question of the trade-off between economic stability and the social needs of the population that the government should address. Some of the previous research in this area has shown that in the late 2000s to early 2010s, the country’s strong position in the international arena and the status of “superpower” was viewed by Russians as quite important and could mitigate to some degree an absence of support for the population from the state. However, by the middle of the 2010s, the situation had changed and Russians started to prioritize the welfare of the general population over a strong stance in the international arena and military power. Moreover, years of economic growth in the 2000s have led to the formation of a population group that does not perceive stability as an a priori value, while the impact of the crisis that started in 2014 has led to an understanding among the population that a focus on stability actually means a focus on the conservation of stagnation without the creation of new “growth points” (Petukhov 2018). All of that means that the mechanism of legitimizing the neoliberal vector in social policy via economic stability and positioning in the international arena is breaking down, which also explains the contradiction between the objective reality and the critical attitudes of the population.

The unfolding epidemiological and resulting economic crisis shatter that stability and are expected to result in a significant reduction in real incomes of the population, a restructuring of the labor market, a change in the balance of labor relations between employers and workers, and a reduction in the number of small- and medium-sized businesses. This could not only reinforce existing inequalities, but also reveal new dimensions (e.g., between those who are able to switch to remote working, use personal transport, and solve their problems with the help of digital technologies in order to protect themselves from the pandemic, and those who are not). In these conditions, the strengthening of negative trends connected with subjective perceptions of inequality is expected, as previous research shows that the problem of inequality does not fade into the background even in times of economic crisis. At the same time, the focus of the state is currently placed mostly on poverty and the prevention of social unrest, not on inequality.

When it comes to developing a socio-economic policy against inequality, one should take into account that income or wealth concentration and monetary inequality among the wider population are different phenomena requiring different control mechanisms. Measures against high wealth concentration include imposing taxes on excess profits, wealth, inheritance, and rent and, in the case of Russia, changing institutional conditions and diversifying the economy to reduce the role of rents in the natural resources sector. The problem of inequality among the mass population can be addressed by redistribution mechanisms (in particular, progressive taxation systems) as well as by ensuring equality of opportunity, first of all in terms of access to education and jobs.

A whole set of measures is needed to get inequality on the meritocratic track. Mechanisms may include systemic changes in the labor market (such as differentiation of the minimum wage according to education level); elimination of regional and industry differences, which are substantial in Russia; and improvement of social protection for households at different life cycle stages (in particular, families with children). Equally important, however, is the acknowledgement of the need for creating a new social contract between society and the state (this understanding is so far absent both among the population and the power elite).

There are many questions that need to be answered when determining the contract’s stance on the problem of inequalities—we will list only some of them. Inequality between which groups is supposed to be redressed? Should measures be limited to supporting the poorest population, should they target inequality between the middle segments of the population, or should they bridge the gap between the upper class and the masses? Should efforts be concentrated on reducing the inequality of opportunity to preserve the stimulating role of inequalities, or does the inequality of outcome (in particular, income) also need to be rectified, as it determines the inequality of opportunity for next generations? Should high-income mobility be promoted or reduced? And how can the scope of “productive” inequality be determined—or are the last two cases not about the scope of mobility and quantitative boundaries of inequality but about their causes and underlying factors? These questions are so far missing from the socio-political agenda; without solving them, however, it is impossible to successfully respond to the inequality challenges and take advantage of the ensuing opportunities in modern Russian society.

## Data Availability

RLMS-HSE data is openly available at https://www.hse.ru/en/rlms/. IS FCTAS RAS data is not available for open use. The author is grateful to the director of the IS FCTAS RAS Mikhail Gorshkov for the access to the data.

## Abbreviations

IS FCTAS RAS: Institute of Sociology, Federal Center of Theoretical and Applied Sociology of the Russian Academy of Sciences
ISSP: International Social Survey Programme
PPP: Purchasing power parity
RLMS-HSE: Russia Longitudinal Monitoring Survey conducted by the Higher School of Economics
RUB: Russian ruble, currency of Russia
WID: World Inequality Database

## Footnotes

1 RLMS-HSE is a series of nationally representative, household-based surveys designed to monitor the effects of Russian reforms on the economic welfare of households and individuals in the Russian Federation. They are conducted by the National Research University “Higher School of Economics” and OOO “Demoscope” together with Carolina Population Center, University of North Carolina at Chapel Hill and the Institute of Sociology of the Federal Center of Theoretical and Applied Sociology of the Russian Academy of Sciences. The RLMS-HSE employs a multi-stage stratified probability sample. The sample size surpassed 12,000 respondents in each wave. A description of the study, the survey schedule, and the data is available at https://www.hse.ru/en/rlms/ and http://www.cpc.unc.edu/projects/rlms-hse.

2 Monitoring by IS FCTAS RAS was a series of nationally representative surveys designed to monitor changes in socio-economic, political, socio-cultural, and ethno-religious spheres of modern Russian society between 2014 and 2018 as part of the “Dynamics of Social Transformation of Modern Russia in Socio-economic, Political, Socio-cultural and Ethno-religious Contexts” project. The monitoring also employed a multi-stage stratified probability sample. The sample size of each wave was 4000 respondents. We also use some of the earlier surveys carried out by IS RAS with the same sampling principles.

3 Data source for PPP: World Bank (http://data.worldbank.org/indicator/PA.NUS.PRVT.PP?locations=RU).

4 Here, we focus only on the economic definition of the middle class, which is based on income and used mostly by economists (Milanovic and Yitzhaki 2002; Kharas 2010; Chauvel 2013, Atkinson and Brandolini 2013; López-Calva and Ortiz-Juarez 2014, etc.). As for the sociological understanding of middle class and contradictions between the middle class and the working class that are much discussed in Western sociology in terms of social mobility and reproducing inequality, they are not articulated in the perceptions of the Russian population and are overshadowed by the contradictions between rich and poor. According to the ISSP 2019 data, 67.9% of Russians declare significant tensions between rich and poor in contemporary Russia (with 29.0% of them considering these tensions to be very strong), while the tensions between the working and middle classes are seen as significant by 23.8% (only 5.9% seeing them as very strong). Moreover, in terms of norms and values related to inequalities, both the middle class and working class share quite similar views. While middle class representatives show the strongest orientation towards initiative, responsibility, and self-reliance, they still share the universal view of the whole population that inequalities in contemporary Russia are too deep and illegitimate, and expect the state to be the main actor in solving this problem, even if they do not rely on the help from the state themselves (Middle Class 2016).

5 Data source: World Bank (http://data.worldbank.org/country/russian-federation).

6 With regard to the general population but not to the top 3–5%, the rich, who are not included in survey samples.

7 The system of social support is vast both on federal and, more importantly, regional levels and covers many different population groups on a categorical basis; however, the means-tested component is small and the absolute levels of many types of monetary benefits are low, making an insignificant input on the income of the population, even for the poor. Categorically based social benefits are one of the legacies of the Soviet model, along with universally provided public services of childcare, education, and healthcare.

8 http://government.ru/projects/selection/741/35675/

9 The European Social Survey, www.europeansocialsurvey.org
